# To the Veterinary Profession at Large

**Published:** 1883-04

**Authors:** 


TO THE VETERINARY PROFESSION AT LARGE.—We wish to call your attention especially to the Case Department. We also wish that every practitioner of the veterinary art in the United States and Canada would make accurate notes of cases of interest falling under his observation and forward the same to us for publication. By so doing the Journal would be a more potent agent in diffusing the practical experience of active veterinarians throughout the country and thus trust to advance and raise the veterinary standard. Trusting that this appeal will not pass unnoticed, we hope to see every State represented in the next issue.

---

THE treatment of fractures in the lower animals has always been looked upon as almost impossible by veterinarians, but this is absolutely wrong. Of late we have been informed by a medical practioner that he has treated six or seven cases occurring below the knee, or hock, by the plaster of Paris splint with perfect success in every case. He applies the plaster splint in the ordinary way, and then turned the animals out to pasture for one or two months. In one case treated in this way in which the fracture occurred just below the knee the animal, after recovery, proved to be the fastest horse in town, which, by the way, was a town in which fast horses were not scarce. These are facts which the veterinarian should consider carefully and put into every day practice if desirous to keep up with this rapidly advancing age.

